# Relationship between the Annealing Temperature and the Presence of PbI_2_ Platelets at the Surfaces of Slot-Die-Coated Triple-Halide Perovskite Thin Films

**DOI:** 10.1021/acsami.3c07692

**Published:** 2023-08-25

**Authors:** Dan R. Wargulski, Ke Xu, Hannes Hempel, Marion A. Flatken, Steve Albrecht, Daniel Abou-Ras

**Affiliations:** † Helmholtz- Zentrum Berlin für Materialien und Energie GmbH, 14109 Berlin, Germany; ‡ Faculty of Electrical Engineering and Computer Science, Technische Universität Berlin, 10587 Berlin, Germany

**Keywords:** halide perovskites, hybrid photovoltaics, scanning electron microscopy, cathodoluminescence, slot-die coating, lead iodide

## Abstract

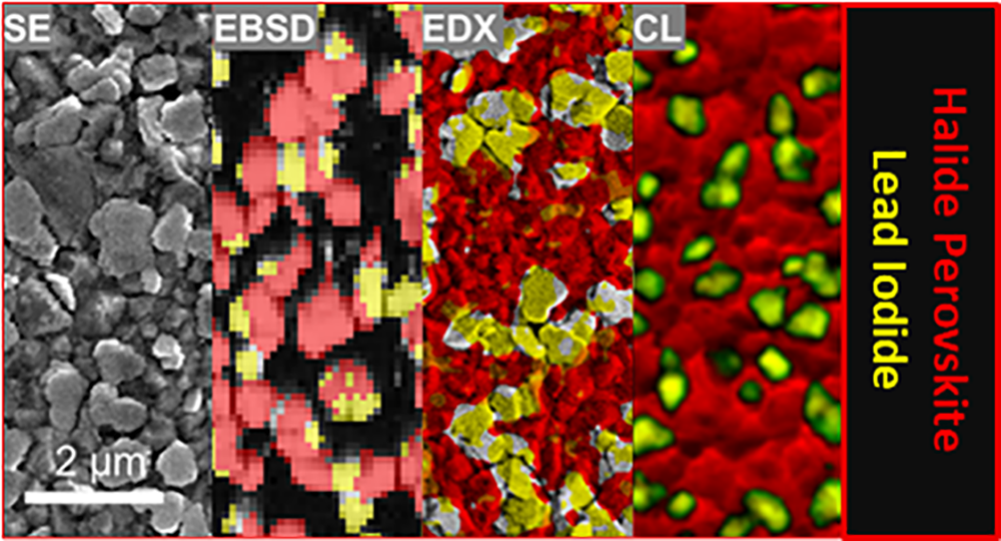

We investigated triple-halide perovskite (THP) absorber layers with 5 mol % MAPbCl_3_ added to the double-halide perovskite (Cs_0.22_FA_0.78_)Pb(I_0.85_Br_0.15_)_3_. As a deposition method, a highly scalable printing technique, slot-die coating, with a subsequent annealing step was used. We found a strong power conversion efficiency (PCE) dependence of the corresponding solar cells on the annealing temperature. The device performance deteriorated when increasing the annealing temperature from 125 to 170 °C, mainly via losses in the open-circuit voltage (*V*_oc_) and in the fill factor (FF). To understand the mechanisms behind this performance loss, extensive characterizations were performed on both, the THP thin films and the completed solar-cell stacks, as a function of annealing temperature. Correlative scanning electron microscopy analyses, i.e., electron backscatter diffraction, energy-dispersive X-ray spectroscopy, and cathodoluminescence, in addition to X-ray diffraction and photoluminescence, confirmed the presence of PbI_2_ platelets on the surface of the THP thin films. Moreover, the area fraction of the PbI_2_ platelets on the film surface increased with increasing annealing temperature. The deteriorated device performance when the annealing temperature is increased from 125 to 170 °C is explained by the increased series resistance and increased interface recombination caused by the PbI_2_ platelets, leading to decreased *V*_oc_ and FF values of the solar-cell devices. Thus, the correlative analyses provided insight into microscopic origins of the efficiency losses.

## Introduction

Triple-halide perovskites (THPs)^1^ are applied as top-cell absorber layers in perovskite-silicon tandem solar cells (PSTSCs). Developed as a wide-band-gap semiconductor,^2^ it can reach the optimum band-gap energy for PSTSC top cells of about 1.7 eV^3^ without the efficiency-limiting, light-induced effect of halide phase segregation.^4,5^

Recently, a record power conversion efficiency (PCE) for PSTSCs of 33.7% was achieved.^6^ However, this record was realized by solar cells fabricated on small device areas via laboratory-scale processes, such as spin-coating. While this technique has shown to be an excellent method to fabricate solar cells on about 1-cm^2^-large substrates, it is rather unsuitable for upscaling, i.e., for the transfer to industrial processes for solar modules. Printing techniques such as slot-die coating can be regarded as promising candidates for the industrial deposition of halide perovskites.

A previous work by Xu et al.^7^ investigated the effect of different annealing temperatures on slot-die-coated THP thin films and solar cells. The aim was to find optimal process parameters and to achieve a suitable crystallization of THP thin films without the application of antisolvents. Therefore, N_2_ quenching was used to dry the deposited ink, followed by an annealing step at various temperatures. It revealed an increasing amount of PbI_2_ with increasing annealing temperatures detected by X-ray diffraction (XRD); however, these PbI_2_ agglomerates were not further investigated, and their impact on the device performance is not discussed in detail.

Therefore, in the present contribution, we continue the work of Xu et al.^7^ by analyzing a series of THP thin films, annealed at temperatures ranging from 100 to 170 °C. Furthermore, completed solar cells from these stacks were investigated. Using various characterization methods in scanning electron microscopy (SEM) in addition to XRD and photoluminescence (PL), it was possible to identify the PbI_2_ precipitates as homogeneously distributed secondary phases and to verify that, indeed, PbI_2_ forms platelets at the THP thin-film surface only. This detailed microscopic insight provided the possibility of proposing a specific model of how the PbI_2_ precipitates affect the photovoltaic parameters of THP solar cells.

## Experimental Section

### Material Synthesis and Device Fabrication

The THP composition studied in the present work was 5 mol % MAPbCl_3_ alloyed to a double-halide perovskite of stoichiometry (Cs_0.22_FA_0.78_)Pb(I_0.85_Br_0.15_)_3_. The THP deposition process consisted of slot-die coating and subsequently N_2_ quenching by a N_2_ knife for controlling thin-film drying, which acts as a replacement of the widely applied antisolvent dripping. The N_2_ quenching followed an annealing step at temperatures of 100, 125, 150, 160, and 170 °C for 20 min.

**Figure 1:**
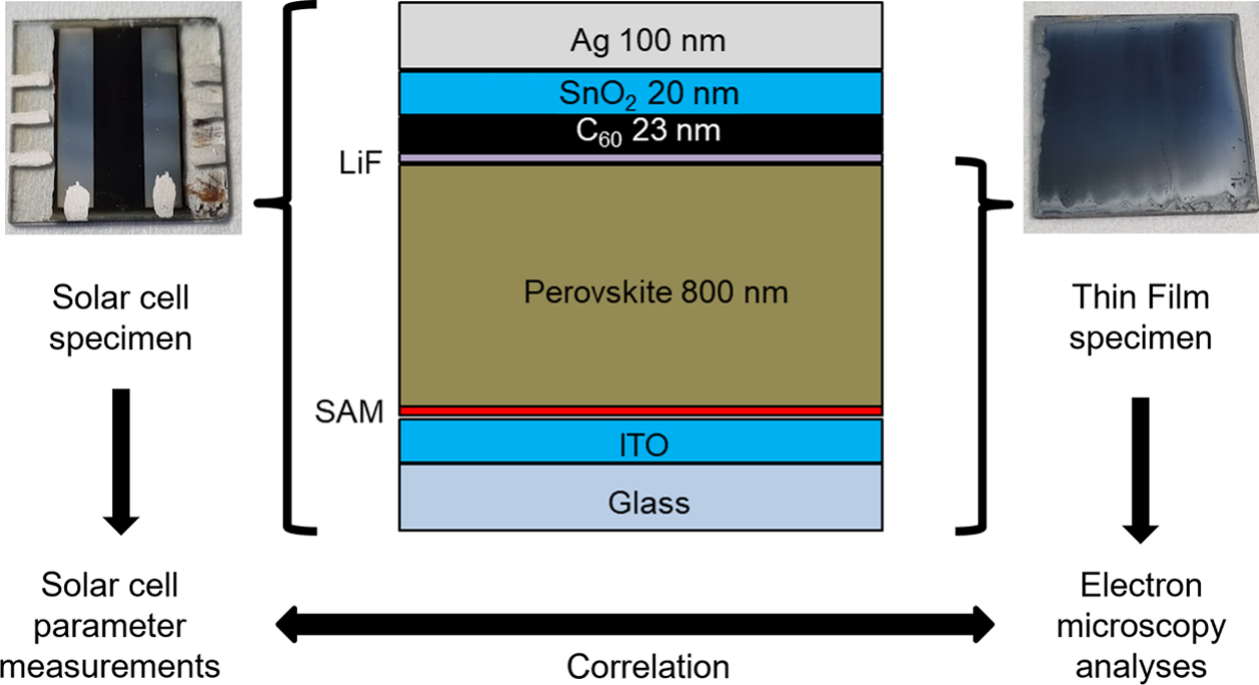
Schematic of the THP solar-cell structure and images of the THP solar-cell specimen made for determination of the solar-cell parameters and the THP thin-film specimen with an open THP surface for electron microscopy analyses.

The THP thin films in the THP/self-assembled monolayer (SAM)/indium–tin oxide (ITO)/glass and Ag/SnO_2_/C_60_/LiF/THP/SAM/ITO/glass stacks were prepared via very similar processes. For complete solar-cell stacks, LiF and C_60_ were thermally evaporated followed by the deposition of SnO_2_. The Ag back-contact was deposited by thermal evaporation. The overall structures of both sample types are depicted in Figure 1. Further details about the materials synthesis, ink preparation, and thin-film deposition are given in the Supporting Information and by Xu et al.^7^

### Solar-Cell and Thin-Film Characterization

The secondary electron (SE) images and cathodoluminescence (CL) mappings were acquired with a Zeiss Merlin scanning electron microscope at an acceleration voltage of 5 kV. The beam current was limited to 50–100 pA to reduce the electron-beam damage of the THP thin films, which can be detected well by reduced luminescence signals. The pixel size of the CL images was set to 27 nm, and the CL intensity was measured with a photomultiplier tube (300 μm dwell time and 1 V gain). In order to obtain monochromatic CL images, 700 ± 50 and 500 ± 50 nm band-pass filters were applied. It was not possible to acquire hyperspectral images by the charge-coupled device due to the rapidly progressing beam damage and the necessarily longer integration time in this measurement mode.

The electron backscatter diffractometry (EBSD) and energy-dispersive X-ray spectroscopy (EDX) measurements were performed by using a Zeiss Ultra Plus scanning electron microscope equipped with Oxford Instruments Symmetry EBSD and Ultim Extreme EDX detectors. The EBSD measurements were conducted at an acceleration voltage of 15 kV. The samples were tilted to an angle of 70° during the measurements. The step size was 50 nm. EDX spectra and elemental distribution maps were acquired at 3.5 kV on nontilted samples.

The solar cells with an active area of about 0.16 cm^2^ were characterized in a sun simulator under 1-sun illumination through the glass substrate. The previous study of Xu et al.^7^ already reported about XRD and PL measurements, which are used and extended in the present work for further analyses and discussion (included in the Supporting Information).

## Results and Discussion

### Analyses of THP Solar Cells

*J*–*V* characteristics of 258 solar cells in total were acquired, and the corresponding solar-cell parameters, i.e., the photoconversion efficiency (PCE), fill factor (FF), short-circuit current density (*J*_sc_), and open-circuit voltage (*V*_oc_), were determined. The data of the 100, 150, and 170 °C samples were extracted from the work by Xu et al.^7^ and added to those of the 125 and 160 °C samples measured for the present study.

**Figure 2:**
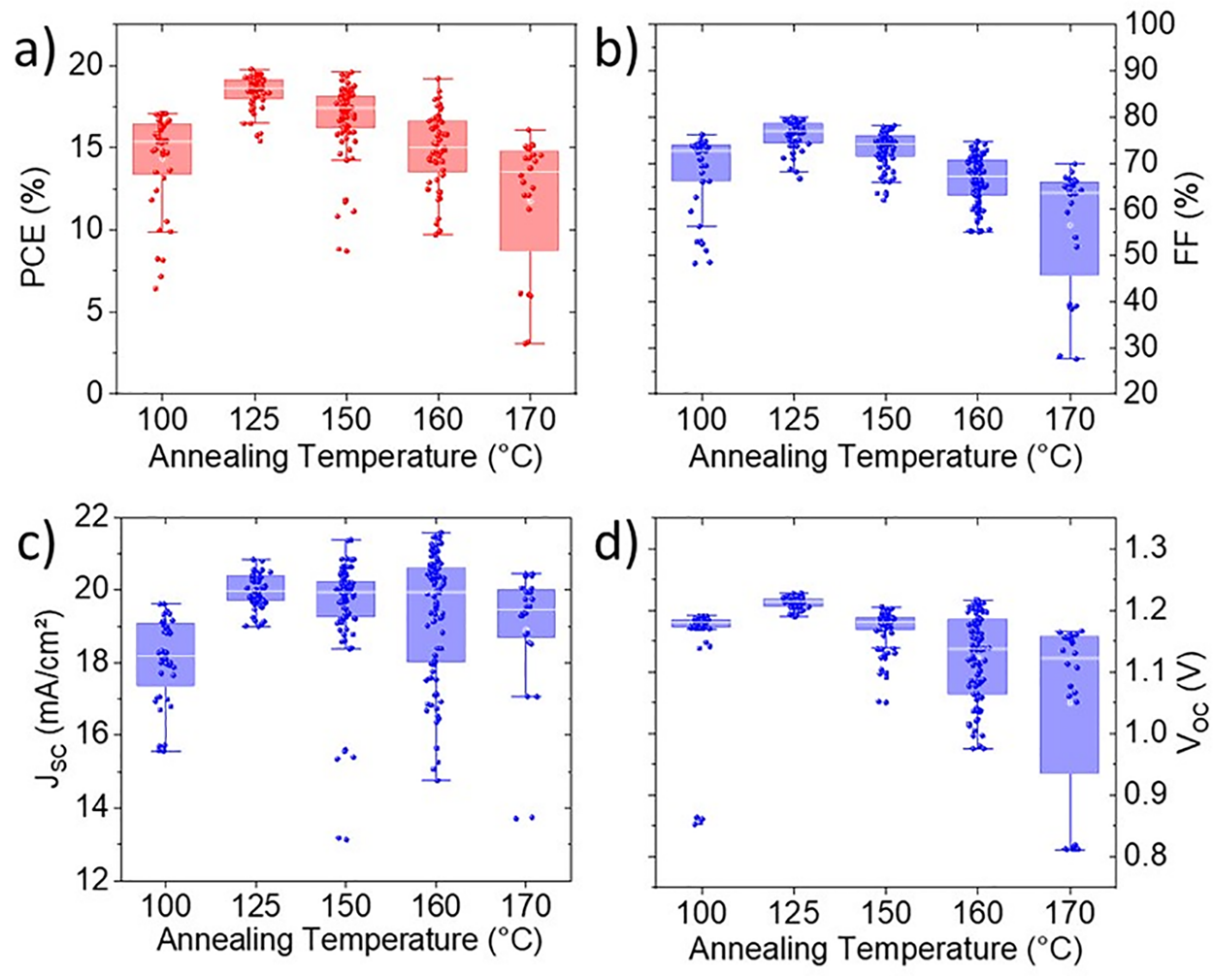
Photovoltaic parameters of the solar cells analyzed in the present work: (a) PCE, (b) FF, (c) *J*_sc_, and (d) *V*_oc_ of solar cells annealed at temperatures between 100 and 170 °C. For PCE, FF, and *V*_oc_, the median values exhibit local maxima at 125 °C annealing temperature and a decrease for higher temperatures.

In the previous study by Xu et al.,^7^ the solar cell with the THP film annealed at 150 °C performed best. However, as shown in Figure 2, it was found in the present work that the maximum PCE peak is found for the 125 °C sample with a value of 19.9% (*J*–*V* measurement shown in Figure S1). The PCE increases from about 15.4% at 100 °C and decreases with increasing annealing temperature for temperatures >125 °C. This maximum value at 125 °C is close to the record PCE of 20.3% reported for single-junction solar cells with slot-die-coated THP absorbers.^1^ The FF as well as the *V*_oc_ values exhibit an increase from 100 to 125 °C followed by a steady decrease for annealing temperatures ranging from 125 to 170 °C. The solar cells annealed at 125 °C exhibited maximum values of FF = 80% and *V*_oc_ = 1.23 V (not in the same solar cell). On the other hand, the median values of *J*_sc_ remain within the interval between about 19.5 and 20 mA/cm^2^. Therefore, the PCE decrease visible between 125 and 170 °C is mainly due to decreases in FF and *V*_oc_.

The irregular parameters of the 100 °C samples indicate insufficient crystallization at such low temperatures. This assumption is further strengthened and discussed in the following results and discussions.

### Microscopic Analyses of the THP Thin Films

**Figure 3:**
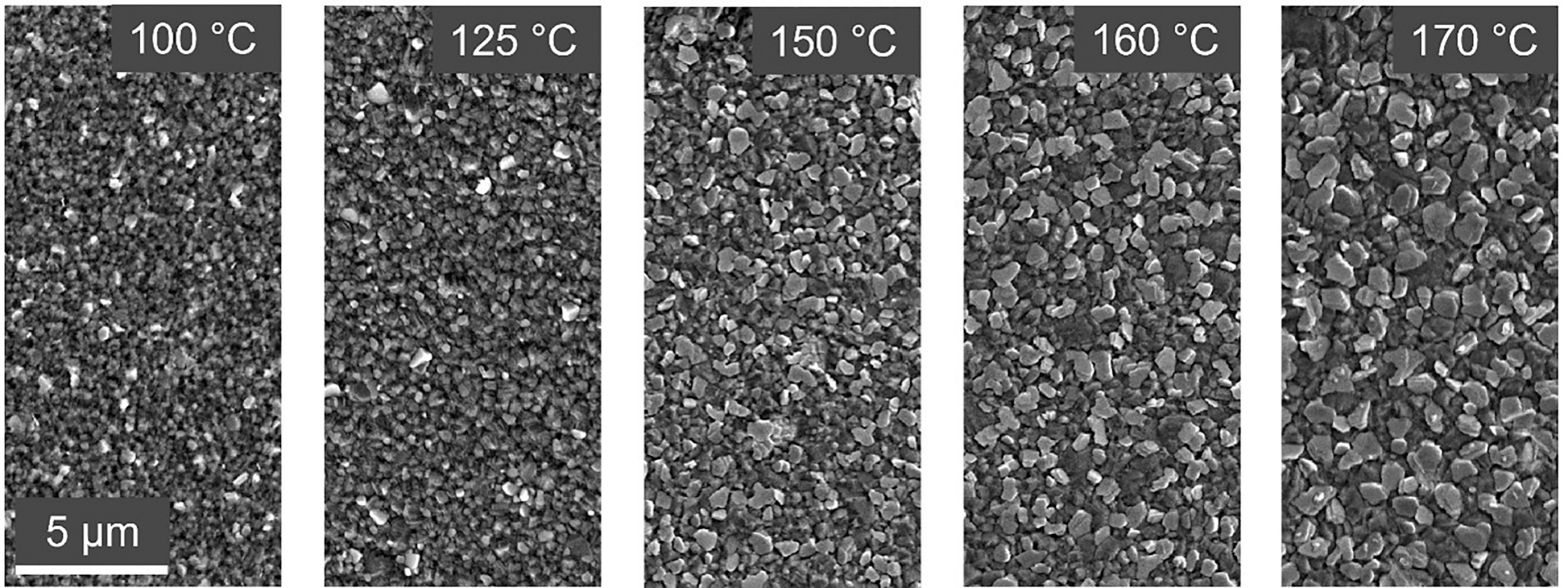
SE images of samples annealed at 100–170 °C showing contiguous THP thin films with increasing crystallite sizes for increasing temperature.

For correlation of the solar-cell parameters and efficiencies with the THP thin-film properties on the submicron scale, various techniques in SEM were applied to THP/SAM/ITO/glass stacks. The SE images of all five samples (Figure 3) show contiguous THP thin films. The sizes of the crystallites increase with increasing annealing temperature, as was already reported for the 100, 150, and 170 °C samples in the previous study by Xu et al.^7^

**Figure 4:**
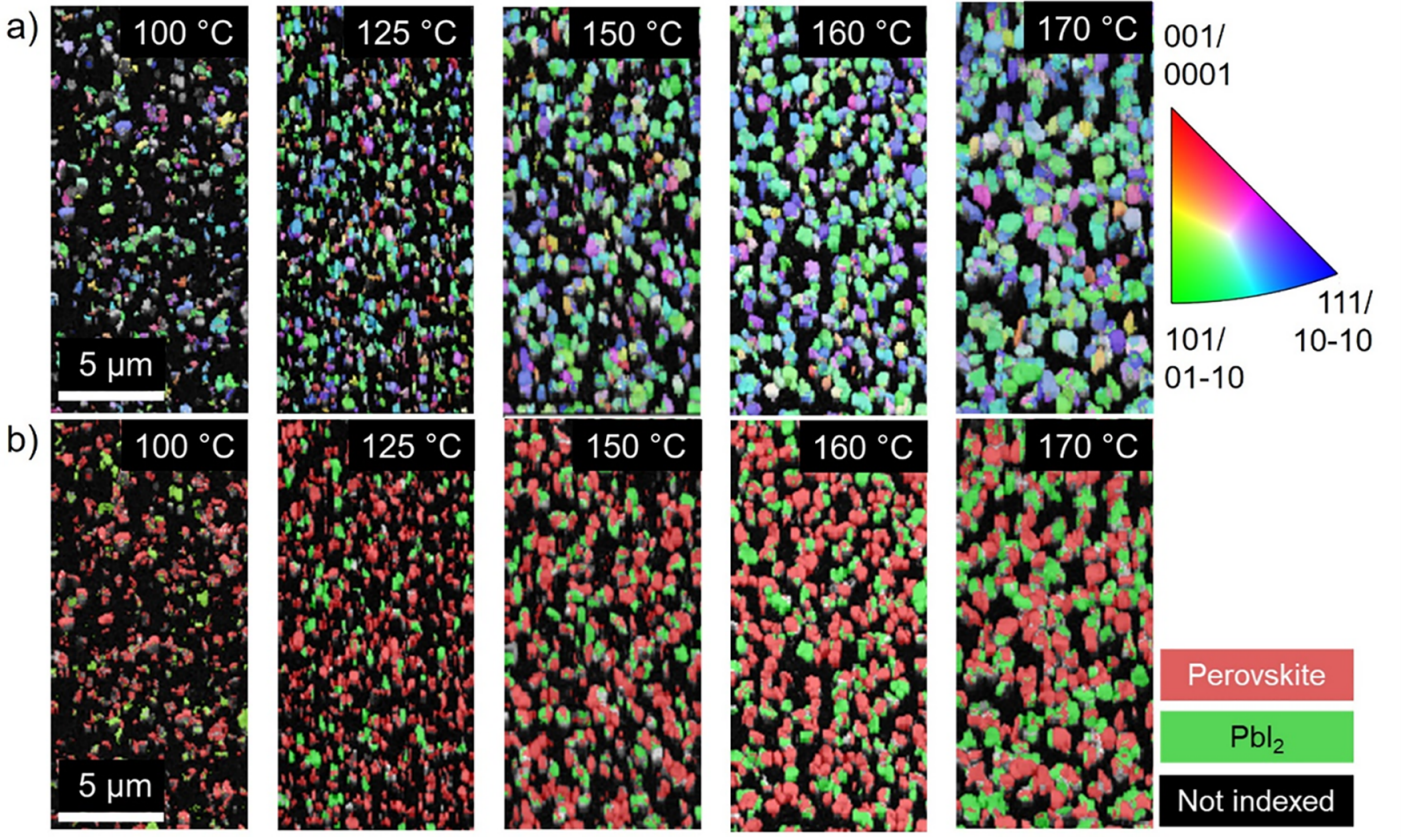
EBSD analyses with (a) the orientation–distribution maps (given by false colors; see the legend with orientations for the pseudocubic THP/trigonal PbI_2_ phases) and (b) the phase distribution maps of the identified THP and PbI_2_ phases.

While SE images are suitable for estimations of grain sizes, EBSD maps provide accurate grain-size and grain-orientation distributions. Figure 4a shows EBSD orientation maps in which different colors correspond to different crystal orientations. The black regions in the maps are those on which the EBSD patterns were not able to be indexed by the software; this may be due to shading via surface roughness and the 70° sample tilt during the measurement or due to grain sizes smaller than the EBSD detection limit.

To analyze the effect of surface roughness of the THP layers with PbI_2_ secondary phases and its effect on the solar-cell performance, we first acquired cross-sectional SEM images (Figures S2 and S3). These images depict the film surface under a small viewing angle and do not exhibit any strong roughnesses. Moreover, it may be assumed that the film roughness affects the coverage of the C_60_ contact layer. However, cross-sectional SEM images and EDX measurements of the completed solar-cell stack (Figure S2) depict a continuous C_60_ layer. It should be noted that, in the present work, the C_60_ layer thickness was about 23 nm, and other publications reported that full coverage of the C_60_ layer can be achieved already at a thickness of 2.3 nm^8^ and that even an ultrathin C_60_ film of about 1 nm thickness can still be functional in terms of efficient solar-cell operation.^9^

The THP thin films exhibit a preferred orientation perpendicular to the {101} crystal planes with no significant differences for the five different temperatures. The increase in the grain size with increasing annealing temperature is visible as well (Figure 4). The corresponding measured grain sizes are provided in Table S1.

From the EBSD data sets, also the phase distributions can be extracted (Figure 4b). Apparently, the thin films consist of the THP phase with fractions of the PbI_2_ precipitates. With increasing annealing temperature, an increase in the area fraction of PbI_2_ crystallites can be detected. The presence of PbI_2_ was already discovered by Xu et al.^7^ and was unexpected because no excess in PbI_2_ was provided during the THP thin-film synthesis. While this previous study provided first hints of an increasing amount of PbI_2_ by XRD and grazing-incidence XRD, the electron microscopy analyses in the present work directly mapped the distributions of PbI_2_ on the THP thin films.

**Figure 5:**
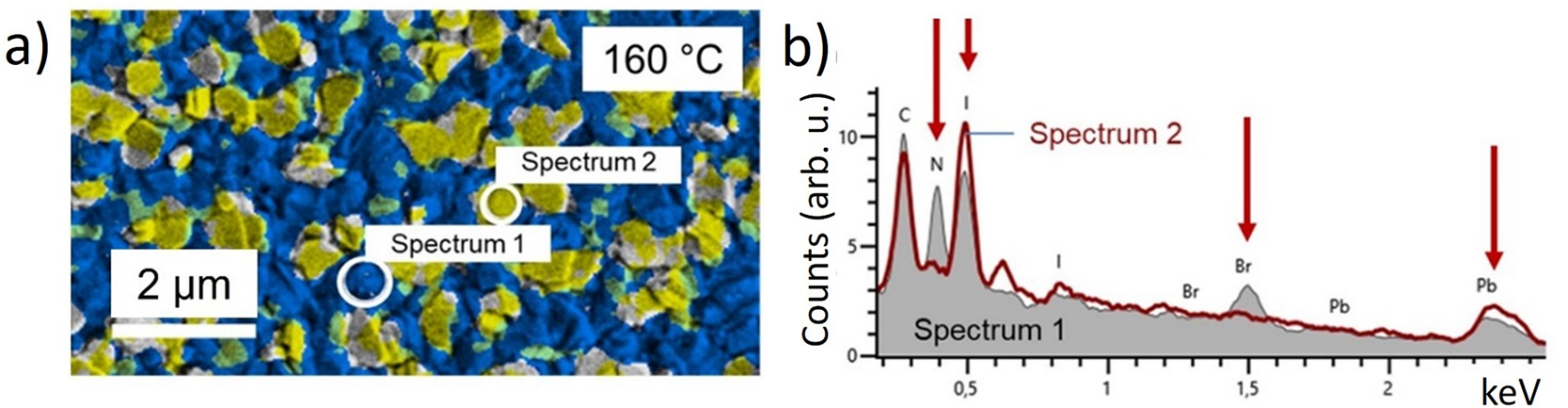
(a) Composite map superimposing the elemental distributions of I (yellow) and N (blue) obtained by EDX (I M and N K X-ray lines) to confirm the presence of PbI_2_ phases. Two different and significantly varying regions in the EDX map were selected (white circles), and the corresponding EDX spectrum 1 (gray filled) and spectrum 2 (red line) extracted and depicted in part b. These spectra revealed that, compared with the THP matrix, the assumed PbI_2_ precipitates are depleted in N and Br and enriched in Pb and I, as expected for PbI_2_.

For confirmation of the phase distributions obtained by EBSD, EDX measurements were applied (Figure 5). However, it was not possible to identify the PbI_2_ precipitates unambiguously in the EDX elemental distribution maps. In these maps, regions were present that exhibited enhanced Pb and I signals, as expected for the PbI_2_ precipitates. The differences in the Pb and I signals between the THP matrix and these regions were too small to be easily detectable in the maps. This fact, also given that rather low electron-beam energies of 3.5 keV lead to low information depths of the measurements, indicates that the extensions of the precipitates in the direction perpendicular to the substrate are very small, about 10–20 nm, which gives rise to the conclusion that the PbI_2_ precipitates are present at the surface as platelets. To highlight the compositional differences between the precipitates and the matrix, the N (blue) and I (yellow) signals are superimposed in Figure 5a. From the regions marked by white circles, EDX spectra were extracted from the THP matrix and the Pb-rich and I-rich regions (Figure 5b). From these two spectra, it is apparent that the precipitates are depleted in N and Br and enriched in Pb and I, as expected for PbI_2_.

**Figure 6:**
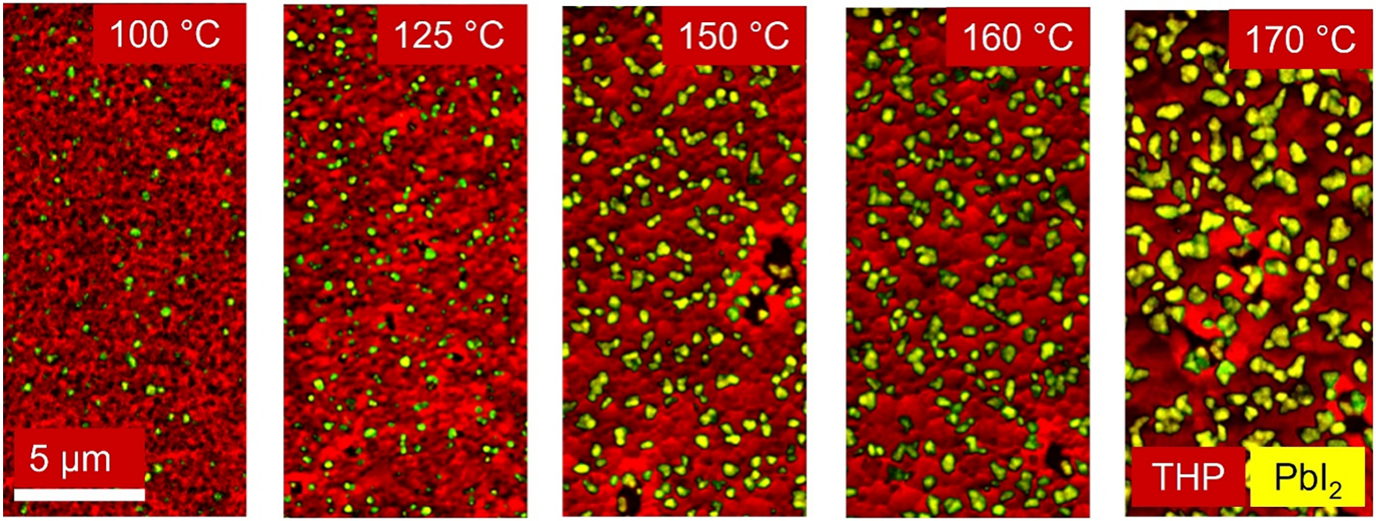
CL composite maps in plane view consisting of the 750 ± 50 nm band-pass filter signal corresponding to the THP luminescence (red) and the 500 ± 50 nm band-pass filter signal corresponding to PbI_2_ (yellow).

CL analyses were able to effectively map the PbI_2_ phases on top of the THP thin films. The main CL peak energy of 1.69 eV (734 nm), expected for the THP phase (Figure S4), agrees well with PL measurements (Figure S5a), and measured peak energies of PbI_2_ at 2.43 eV (511 nm) are in the expected range as well.^1^ By the superimposition of monochromatic CL images acquired using band-pass filters of 750 ± 50 and 500 ± 50 nm, detailed false-color maps of the THP and PbI_2_ phase distributions were obtained (Figure 6). These CL images reveal the area coverage of the PbI_2_ precipitates on top of the THP thin films. The two phases were very distinguishable, which allowed for a quantification of the results employing image segmentation and further analyses of the PbI_2_ coverage.

**Figure 7:**
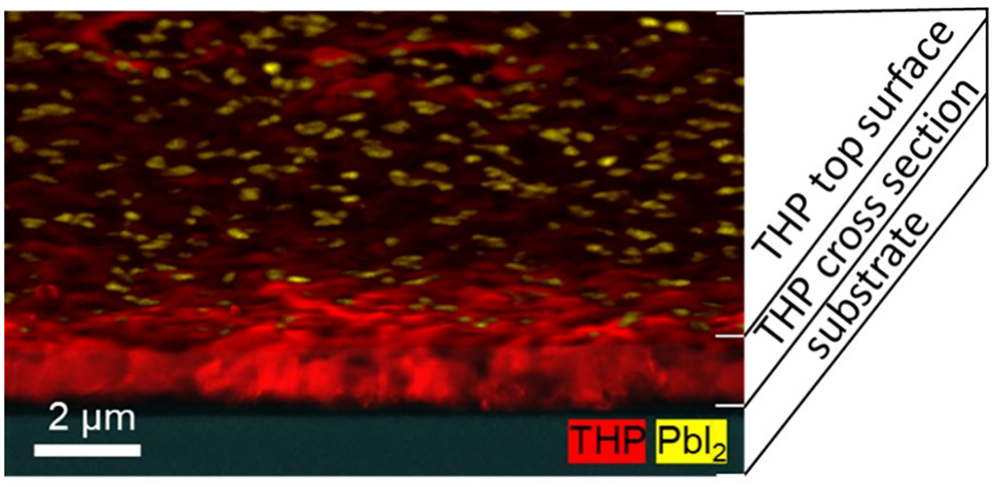
Cross-sectional CL composite map consisting of the 750 ± 50 nm band-pass filter signal including the THP luminescence (red) and the 500 ± 50 nm band-pass filter signal for PbI_2_ (yellow).

The cross-sectional CL image in Figure 7, in which once more the PbI_2_ (yellow) and THP (red) phase distributions are depicted superimposed on the SEM image, shows the PbI_2_ covered surface of the thin film without any PbI_2_ precipitates detectable within the bulk of the THP thin film. This result confirms that PbI_2_ precipitates form as platelets only on the film surface during the annealing process. If PbI_2_ precipitates were existent in considerable amounts in the bulk, it would be easily revealed by microscopic investigations in the cross section, as shown by other reports.^10^ However, no PbI_2_ was detected in the THP bulk by EDX measurements on cross-sectional specimens prepared from solar-cell stacks (Figure S2).

### Influence of PbI_2_ Coverage on FF and *R*_s_

A common reason for low FF values is the high series resistance *R*_s_ of the solar cell. The increasing area fraction of PbI_2_ can increase *R*_s_, which strongly influences the FF.^11^ The shunt resistance was analyzed as well; however, the determined median values of 0.6–2.4 kΩ·cm^2^ did not indicate any shunting or other deterioration of the photovoltaic performance (Figure S6b).

**Figure 8:**
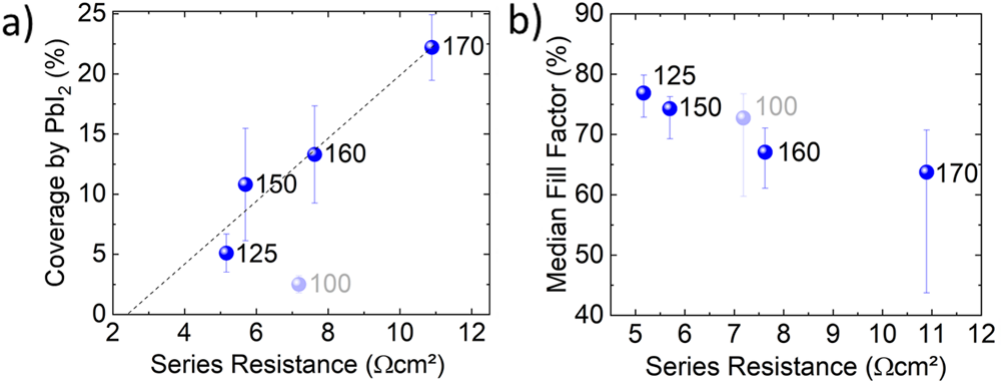
(a) Estimated PbI_2_ coverage as a function of the median series resistance *R*_s_ with a guide for the eyes (dashed line) showing a theoretical PbI_2_-free *R*_s_ of 2.5 Ω·cm^2^ at the *x* intercept and (b) median FF as a function of the median *R*_s_. Data points are labeled with their corresponding annealing temperatures in degrees Celsius. The 100 °C line is illustrated in light gray to mark it as an outlier.

Figure 8a shows the PbI_2_ thin-film coverage plotted as a function of the median series resistance of the measured solar cells. The data of the coverage were extracted from the CL images by image segmentation, while the *R*_s_ data originated from the measured *J*–*V* curves of the solar cells. The viewgraph in Figure 8a reveals a quasilinear correlation between PbI_2_ and the median *R*_s_, for all temperatures except for 100 °C. This linear relationship agrees with an assumption of a decrease in the conductor cross section with increasing PbI_2_ coverage. The coverage is about 3% at 100 °C and reaches about 22% at 170 °C. *R*_s_ exhibits a higher value of 7 Ω·cm^2^ at 100 °C, decreases to its minimum of 5 Ω·cm^2^ at 125 °C, and increases to 11 Ω·cm^2^ at 170 °C. To check the *R*_s_ values for consistency, FF and *R*_s_ were plotted (Figure 8b) and should follow the approximately linear behavior as described by Green’s empirical expression:^12^
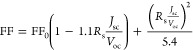
1where FF_0_ corresponds to a fill factor without the influence of *R*_s_. For large characteristic resistances  compared with *R*_s_, it can be approximately described as linear, as shown in Figure 8b. The 100 °C sample and its median *R*_s_ stand out due to its insufficient film crystallization, which makes it a poor semiconductor without the influence of PbI_2_ secondary phases.

### Influence of PbI_2_ on *V*_oc_

**Figure 9:**
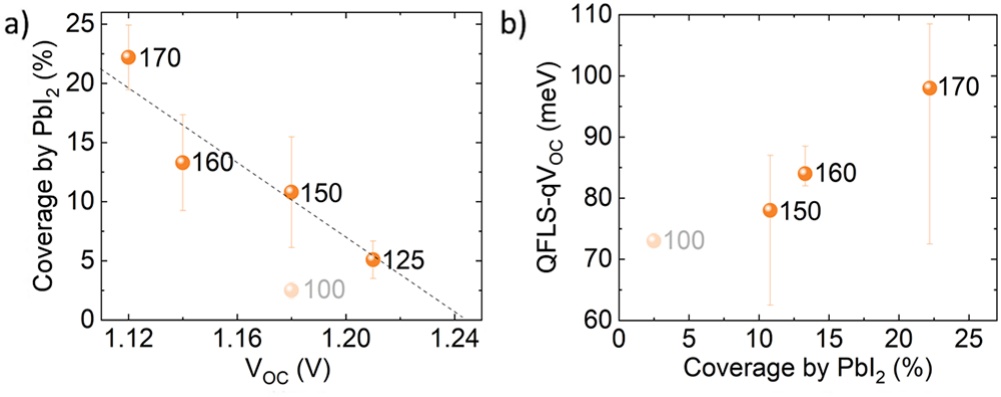
(a) Estimated PbI_2_ coverage as a function of the median *V*_oc_. The dashed line corresponds to a guide for the eye, pointing to a hypothetical *V*_oc_ without any PbI_2_ coverage. (b) Mismatch between QFLS and median *V*_oc_ as a function of the PbI_2_ coverage for the annealing temperatures 100, 150, 160, and 170 °C (data for 125 °C were not available), indicating increasing recombination losses with increasing amounts of PbI_2_.

*V*_oc_ decreases as well with an increase in the annealing temperature (Figure 2b). Figure 9a indicates a linear correlation between the thin-film coverage by PbI_2_ and a decrease in the median *V*_oc_ values. The 100 °C data point is an outlier again. The linear regression reveals a potential PbI_2_-free median *V*_oc_ of about 1.25 V. Describing the exact correlation between recombination and the amount of PbI_2_ on the surface of the THP thin film is more complex than referring only to the correlation between *R*_s_ and PbI_2_. This is because PbI_2_ may have passivating properties^13,14^ in appropriate amounts and at corresponding locations, and complete removal may potentially reduce surface passivation or even generate new recombination centers. Additionally, the band-gap energy decreases with increasing annealing temperature, as indicated by mean measured PL peak energies ranging from 1.67 to 1.65 eV (Figure S5). This result can be attributed to a loss of chlorine, which leads to a further decrease of *V*_oc_. Such an issue is independent of PbI_2_ and difficult to avoid but plays only a minor role in the temperature-dependent *V*_oc_ decrease.

Figure 9b shows the mismatch between quasi-Fermi-level splitting (QFLS) and the measured *V*_oc_, indicating increasing recombination losses with increasing THP thin-film coverage by PbI_2_ platelets. The effective lifetimes obtained from time-resolved photoluminescence (TRPL) measurements (Figure S7) on the THP thin films without an electron-transport layer (ETL; Table S2) are about the same order of magnitude (about 300 ± 100 ns) for the entire temperature range. Thus, we neglected any strong contributions of the THP bulk properties to the performance losses found with the annealing temperature. Rather, elevated recombination rates at the THP/C_60_ interface in connection with the PbI_2_ platelets must be considered.

### Formation of PbI_2_ and Its Effects on the Solar Cell Performance

The formation of PbI_2_ in slot-die-coated THP films is not a result of differences in the chemical compositions already in the inks but of compositional changes occurring in the fabricated THP films after the annealing process. The elevated annealing temperatures of 100–170 °C, compared with lower temperatures in antisolvent-quenched spin-coating film depositions, lead to an increased evaporation rate of organic compounds in the THP ink during the annealing step and finally to a collapse of the ABX_3_ perovskite structure.

In the past, when investigating the synthesis of the mixed-halide perovskite MAPbI_3–*x*_Cl_*x*_ was very popular, a material that in most cases did not contain chloride in detectable amounts, it was found that annealing temperatures above 100 °C led to significant losses of MACl.^15,16^ The same can also be assumed for FACl in THPs containing FA and Cl. A study conducted by Song et al.^17^ showed how volatile compounds such as FACl and MACl influence the stoichiometric ratios in gas-quenched (without the application of antisolvents) perovskite absorber layers by different annealing parameters. It is assumed that FACl/MACl losses lead to Pb-rich surfaces, eventually resulting in the formation of PbI_2_ on the film surfaces. While Song et al.^17^ correlated the thin-film stoichiometry with the device performances, these authors did not link the impaired device performances and stabilities with the amount of PbI_2_. Guo et al.^18^ explained the presence of PbI_2_ at the film surface by the migration of excess PbI_2_ to material interfaces.

An indication for chloride losses in the THP films studied in the present work was found by means of PL. The PL peak energies (Figure S5a) decreased from 1.67 to 1.65 eV for annealing temperatures increasing from 150 to 170 °C. However, this difference is not sufficient to explain the performance losses of the corresponding solar cells.

Several authors reported that an increase of PbI_2_ on the surface of perovskite thin films can be beneficial for the solar-cell performance,^13,14,19−22^ due to passivation of the interfaces^13,14^ but also due to important influences on the thin-film formation and crystallization.^22^ In this study, the detrimental effects of PbI_2_ clearly predominate. One reason for this fact is that the solar-cell structure in the present work differs from most of these reports. In studies and publications dealing with regular solar-cell structures, PbI_2_ was generally situated between the halide–perovskite absorber layer and hole-transport layer.

**Figure 10:**
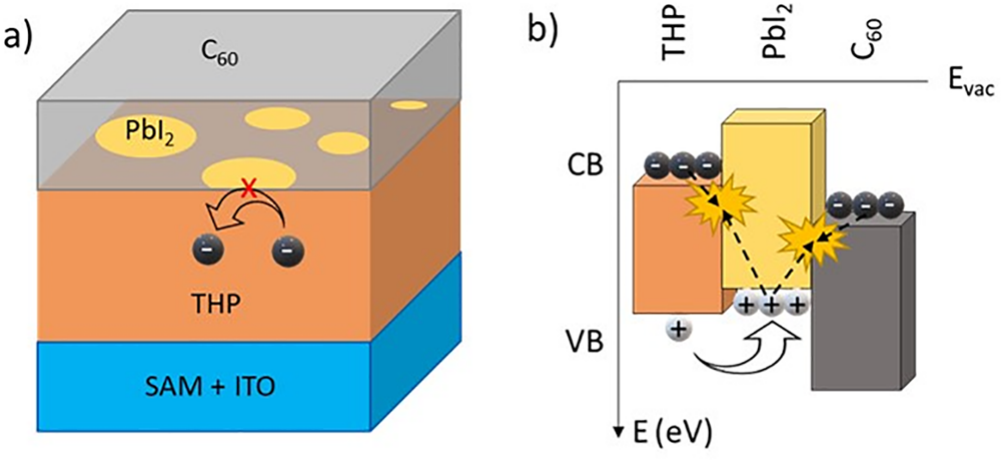
(a) Schematic of PbI_2_ platelets situated at the THP/C_60_ interface in the ITO/SAM/THP/C_60_ stack, a part of an inverted THP solar cell, and (b) schematic band diagram depicting the detrimental effects of PbI_2_ at the THP/C_60_ interface on the charge-carrier-transport and recombination processes.

For integration of the THP solar cell as the top cell in a tandem device,^23^ the “inverted” perovskite solar-cell structure was used in the present work. In this inverted structure, PbI_2_ is located at the interface between THP and the ETL, as shown in Figure 10a. The detrimental effect of PbI_2_ in this structure is a consequence of its elevated conduction band edge compared with those of perovskite compounds usually applied in solar cells. This fact explains why PbI_2_ may block photogenerated electrons from THP and inhibits transfer to the ETL.^24−27^ Nevertheless, even the alignment of the perovskite–PbI_2_ heterojunction is a matter of debate.^22^ We assume an electron-blocking behavior by forming a type 1 alignment^26^ or type 2 alignment (Figure 10) with hole-injecting properties.^25^ Both alignment types are detrimental in inverse THP solar-cell structures.

There are indeed reports claiming beneficial effects even at the absorber–ETL interface; however, they are about the passivation of TiO_2_, which was not applied in the present work.^13^ Instead, we integrated LiF-passivated C_60_ as a selective ETL. The interface between this ETL and PbI_2_ is not yet well investigated. Regarding long-term stability, PbI_2_ is widely seen as a degradation accelerator and reduces the device stability.^28−31^ We have not analyzed the long-term stability, but we assume a stability-harming effect of PbI_2_ in our samples.

It should be noted that the detrimental effect of PbI_2_ is not an issue exclusively in THP solar cells. It can be generalized to all perovskite-type solar cells with inverted cell structure, containing an absorber with a conduction band edge of several 100 meV lower than that of PbI_2_^24^ and with a suitable amount of PbI_2_ present at the absorber–ETL interface.

### Correlation of the Microstructure and Solar-Cell Performances

The experimental results revealed the 100 °C sample as an outlier in the measurements of the solar-cell parameters. Treating it as an outlier is based on the results and conclusions of XRD (Figure S8) and EBSD measurements (Figure 4). In the XRD patterns, the (100) Bragg diffraction peak of the THP at around 14.2° of the 100 °C sample exhibits the largest full width at half-maximum compared with those of the other samples. Additionally, the large fraction of nonindexed areas in EBSD mappings are an indication for regions with grain sizes below the detection limit of the EBSD system. Such small grain sizes correspond to high grain-boundary densities with the result of lower charge-carrier mobilities and increased recombination losses. Thin-film formation at lower temperatures, such as 100 °C, is usually promoted and enhanced by the application of antisolvents in spin coating, but for large-area depositions and in future roll-to-roll processes, antisolvents cannot be sufficiently supplied.^7,32^ Annealing temperatures of 100 °C are not sufficient to form grains of suitable sizes, and even leftovers of intermediate phases could be speculated; therefore, the 100 °C samples are excluded from following analyses and discussions. The inability to use antisolvents makes the annealing temperature one of the most important parameters for perovskite thin-film crystallization in slot-die coating. We consider an annealing temperature of 125 °C as the lower limit, where a desired crystallization can be expected without the application of antisolvents.

The goal of the correlation between the THP thin-film microstructure measured by techniques of electron microscopy and the solar-cell performance parameter is to understand the mechanisms behind the evolution of solar-cell parameters with increasing annealing temperatures. The electron microscopy analyses revealed two main trends: temperature-dependent grain sizes and temperature-dependent area fractions of PbI_2_ platelets on the surfaces of the THP thin films. Furthermore, the solar-cell parameters exhibit the following trends with increasing annealing temperature: no significant change in the *J*_sc_ values and decreases in *V*_oc_, FF, and (as a result of these trends) PCE.

The measured increase in the grain size is considered to be a beneficial influence on the solar-cell performance because grain boundaries are commonly known as locations of increased nonradiative recombination.^33^ Grain boundaries can scatter charge carriers and, finally, decrease their mobility. Larger grain sizes are equivalent to a lower grain-boundary density in the bulk material, and as a result, the bulk recombination decreases. Lower recombination rates and high charge-carrier mobility lead to a higher amount of collectable charge carriers and finally should increase *J*_sc_, which we did not find. The same positive effects are expected for the *V*_oc_ value because it is a parameter connected to recombination processes in solar cells. An increase in recombination losses with increasing annealing temperature can be regarded as the main reason for low *V*_oc_ values. Therefore, another presumably PbI_2_-related mechanism rules the recombination processes, opposing the beneficial effect of increasing grain sizes.

Analyses of the PL measurements provided values for QFLS in the THP thin films. Such QFLS values represent the upper limit of *qV*_oc_. A mismatch between QFLS and *qV*_oc_ indicates losses due to recombination processes, which seem to increase strongly with increasing annealing temperature (Figure 9b). The reason for the increasing recombination rates might be the opening of new recombination pathways by PbI_2_ (Figure 10b). Holes can enter PbI_2_ and potentially recombine with blocked electrons in the THP or electrons in the C_60_ ETL.

The blocking of electrons by PbI_2_ explains the increase in *R*_s_ with increasing PbI_2_ coverage. The linear correlation between the PbI_2_ coverage and *R*_s_ allows a linear approximation of a hypothetical *R*_s_ of PbI_2_-free solar cells (Figure 8). As a result, a PbI_2_-free *R*_s_ of 2.5 Ω·cm^2^ was determined. The measured values for FF and PCE in Figure 2 can be recalculated with the hypothetical PbI_2_-free *R*_s_ using Green’s equation (eq 1) to model the potential of complete PbI_2_ removal.

**Figure 11:**
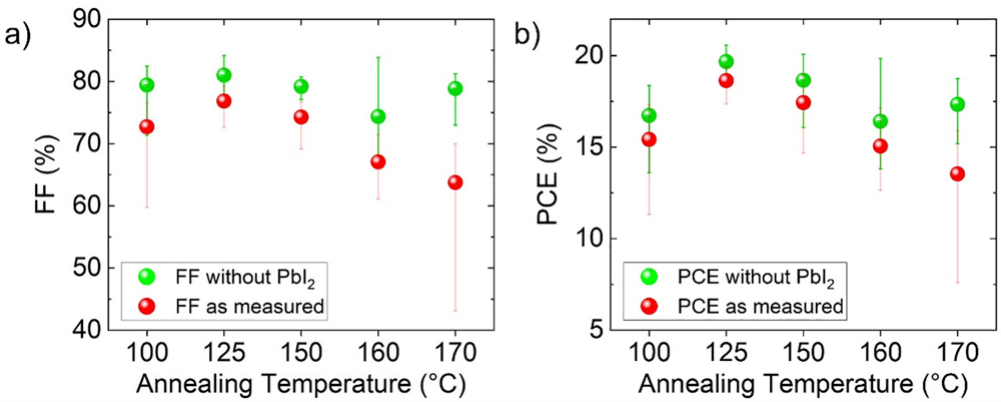
Simulated data of (a) FF and (b) PCE with the subtracted influence of PbI_2_ by recalculating FF and PCE using eq 1 with an approximated median *R*_s_ for a 0% PbI_2_ thin-film coverage of 2.5 Ω·cm^2^. The median gain in the PCE was calculated to be about 1–2%. For comparison, the measured median values are included in red.

The simulated effect of PbI_2_ removal is shown in Figure 11. It reveals an overall increase of FF (Figure 11a), median values exceeding 80%, and median PCEs reaching almost 20% (Figure 11b), as well as a flattening of the decreasing trend with increasing annealing temperatures. The comparison with measured mean PCE values exhibits that all, but especially the 170 °C sample, would benefit from such a removal. The potential gain of PbI_2_ removal regarding the PCE is at least 1%. In a similar way, a PbI_2_-free *V*_oc_ of more than 1.24 V can be determined and would double the PCE gain to more than 2%. Attempts to remove PbI_2_ have already been done by polishing or chemical washout,^34,35^ but complete removal has not been achieved yet.

Notably, the presented model requires the entire removal of PbI_2_, which is not easily done. Such a removal may even lead to a lack of PbI_2_ and the potential opening of new recombination pathways because the presence of PbI_2_ has been reported to enhance passivation.^13,14^ Another potential effect missing in the present model is a presumable increase in *J*_sc_ with increasing annealing temperature due to the increase in the grain size, reducing grain-boundary recombination and due to enhanced collection by reducing the PbI_2_ interface recombination.

## Conclusion

The present study showed that the PCE of solar cells with slot-die-coated THP absorbers is limited by increased area fractions of PbI_2_ precipitates on the THP thin-film surface with increased annealing temperature. By means of correlative electron microscopy, we demonstrated that, while a temperature of 100 °C is not sufficient without the application of antisolvents, the samples annealed at temperatures above 125 °C revealed a correlation between the PbI_2_ coverage and photovoltaic parameters. The stronger coverage of the THP thin-film surface by PbI_2_ precipitates causes a significant increase in the series resistance *R*_s_ of the corresponding solar cells, which again is responsible for the decreasing FF values. Moreover, the PbI_2_ precipitates give rise to recombination pathways at the interfaces, leading to *V*_oc_ losses. These detrimental effects of PbI_2_ are even more effective in inverted perovskite solar-cell structures due to its accumulation at the THP–ETL interface.

We estimated that complete removal of PbI_2_ by chemical polishing or washing will lead to an absolute PCE gain of at least 1%. This removal would prevent higher series resistances and recombination pathways induced by the PbI_2_ precipitates and allow enhanced device performance at higher annealing temperatures via larger average grain sizes.

## Supporting Information Available

Experimental details of materials synthesis and sample preparation and additional results, tables of EBSD data and TRPL results, and figures of CL spectra, PL data, XRD data, series and shunt resistance data, EDX data of solar-cell cross sections, and SE images of cross sections (PDF)
