# Supplementary material for: Relationship between the Annealing Temperature and the Presence of PbI2 Platelets at the Surfaces of Slot-Die-Coated Triple-Halide Perovskite Thin Films
Source: ACS Appl Mater Interfaces. 2023 Aug 25;15(35):41516–24. doi: 10.1021/acsami.3c07692 (PMC10485798; doi:10.1021/acsami.3c07692)
Supplement: Supplementary file 1 — am3c07692_si_001.pdf [file am3c07692_si_001.pdf]

Supporting Information

Relationship between the Annealing Temperature and the Presence of PbI_2 Platelets at the Surfaces of Slot-Die-Coated Triple-Halide Perovskite Thin Films

Dan R. Wargulski[1*], *Ke Xu*[1], *Hannes Hempel*[1], *Marion A. Flatken*[1], *Steve Albrecht*[1,2] *and Daniel Abou-Ras*[1*]

[1] Helmholtz- Zentrum Berlin für Materialien und Energie GmbH, 14109 Berlin, Germany

[2] Faculty of Electrical Engineering and Computer Science, Technische Universität Berlin, 10587 Berlin, Germany

[*]Email: dan.wargulski@helmholtz-berlin.de

[*]Email: daniel.abou-ras@helmholtz-berlin.de

This PDF file includes:

Material Synthesis and Sample Preparation

Additional Results and Measurements

Table S1 to S2

Figure S1 to S9

References

Material Synthesis and Sample Preparation:

The double-halide perovskite $(Cs_{0.22}FA_{0.78})Pb(I_{0.85}Br_{0.15})_3$ was synthesized by mixing CsI (Cesium Iodide 99.999%, abcr GmbH), FAI (formamidinium iodide 99.9%, Dyenamo), PbI_2 (Lead Iodide 99.99%, TCI) and $PbBr_2$ (Lead Bromide 99.99%, TCI) in the ratio of its stoichiometry without using excess PbI_2. The $MAPbCl_3$ was a product of mixing MACl (Methylamonium chloride 99%, Greatcell Solar) and $PbCl_2$ (Lead Chloride, 99%, TCI) in a 1:1 molar ratio. To prepare the ink for the slot-die coating the resulting perovskite salts have been mixed in a molar ratio of 1:0.05 and diluted in a 1.4 mol/ml concentration in a mixed solvent consisting of 93% DMF (Dimethylformamide 99.8%, Sigma Aldrich) and 7% NMP (1-Methyl-2-pyrrolidinone 99.5%, Sigma Aldrich).

As substrate an ITO-covered glass was used. All substrates were cleaned in successively carried ultrasonic bathes in Mucasol solution, DI-water, acetone, and isopropanol for 15 minutes each. The SAM (2PACz, TCI) hole-transport layer was applied by spin coating (5s acceleration to 3000 rpm, 3000 rpm for 25 s) a SAM-ethanol solution (1 mg/ml) after an UV-O3 cleaning step. 10 minutes of heating at 100 °C results in a 1 nm SAM layer.

The slot-die-coating machine used for THP deposition was a FOM ALPHASC from FOM Technologies and the air knife a model 10012XHA from Nex Flow. The substrates were coated with a speed of 30 mm/min and supplied with ink by a rate of 300 µl/min at 21 °C.

Additional Results and Measurements:

Table S1 shows the quantification of the EBSD measurement. The mean grain sizes were determined for both phases. Grain sizes are defined here as circle equivalent diameter. The mean grain size was calculated in two ways, arithmetically and area weighted. The THP grain size increases up to 0.69/0.82 µm at the highest annealing temperature. The mean grain size values for the 100 °C samples are strongly affected by EBSD detection limits. The 100 °C sample exhibits regions of small grains with sizes below the detection limit (212 nm with 50 nm pixel size), corresponding to a large amount of the non-indexed black areas in the EBSD maps. Since these small grains are missing in the calculation, the mean values are increasingly overestimated as higher the share of grains is with sizes below 212 nm. That the mean grain sizes of the 100 °C samples are indeed smaller than the grains of the 125 °C samples, in contrast to what is shown in **Table S1**, is indicated by SE images and the XRD measurements.

Table S1: Grain size measurements by EBSD showing the arithmetic and area weighted mean grain sizes of the THP phase and PbI_2 crystallites.

Sample	Anneal. time (min.)	Grain size (arith. mean) (µm)	Grain size (area weighted mean) (µm)	PbI_2 Grain size (arith. mean) (µm)	PbI_2 Grain size (area weighted mean) (µm)
**100 °C**	20	0.53 ± 0.02	0.56 ± 0.02	0.56 ± 0.03	0.61 ± 0.03
**125 °C**	20	0.52 ± 0.01	0.54 ± 0.01	0.55 ± 0.02	0.58 ± 0.02
**150 °C**	20	0.58 ± 0.03	0.63 ± 0.03	0.54 ± 0.02	0.58 ± 0.02
**160 °C**	20	0.61 ± 0.04	0.68 ± 0.04	0.56 ± 0.03	0.61 ± 0.03
**170 °C**	20	0.69 ± 0.07	0.82 ± 0.07	0.59 ± 0.04	0.67 ± 0.04

The best performing solar cells were determined among the 125 °C samples. A *J-V* measurement with forward and reverse scan is shown in **Figure S2** with the corresponding solar-cell parameters.

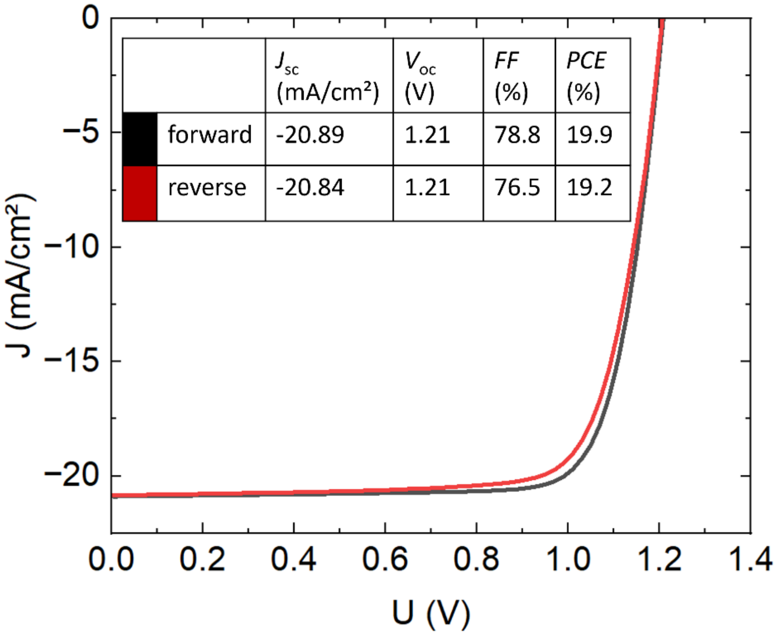

Figure S1: J-V measurements with forward (black) and reverse (red) scan of the best performing 125 °C device with an inlet showing the determined solar-cell parameters.

To exclude another detrimental influence or cause for the decreasing solar cell performances at increasing annealing temperatures than the amount and coverage by PbI_2, the states of the various solar-cell layers such as the THP covering C_{60} were inspected by SE imaging and EDX mapping. The EBSD analyses has revealed non-indexed areas which could be caused by increased surface roughness. One could assume a very high roughness could cause issues regarding the full coverage of the THP by C_{60}. The SE cross-section image in **Figure S2a** shows a 150 °C solar-cell sample with the easy distinguishable layers of the substrate, the ITO electrode, THP thin film and the Ag electrode. The THP surface does not show a roughness which could not be covered by C_{60} ALD deposition. An EDX mapping of the carbon distribution from the same area reveals the 23 nm thin C_{60} which appears continuous and without faults in **Figure S2b**. There were no PbI_2 crystallites or particles visible in the SE image. EDX mappings of Pb and I agree with it and do not reveal any PbI_2 indicating contrasts (**Figure S2c-d**).

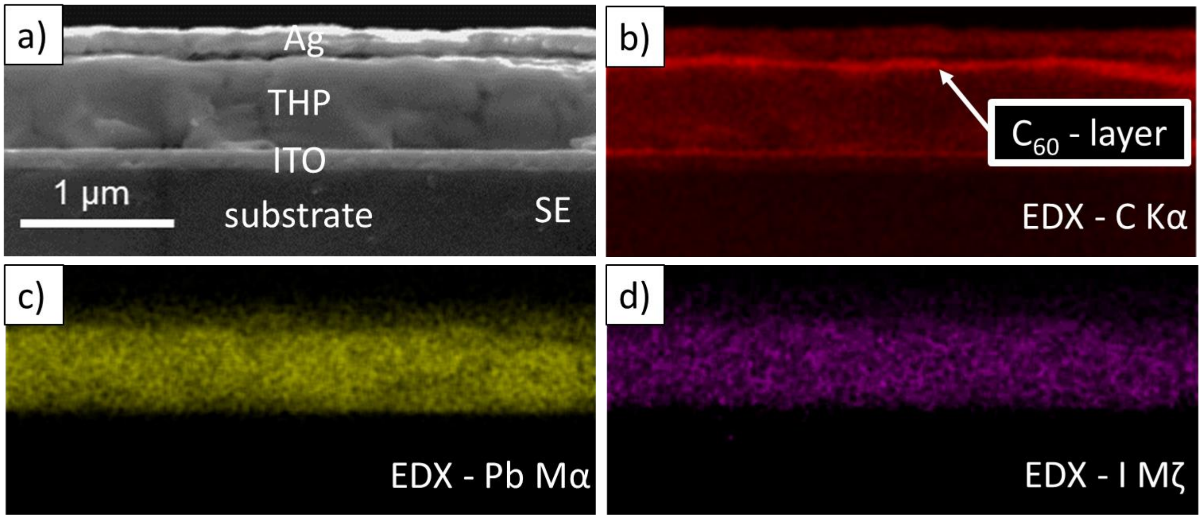

Figure S2: a) SE image of the solar cell cross section (150 °C) with visible layers of the Ag and ITO electrodes and the thicker THP, b) EDX map of the carbon Kα signal from the same area showing the continuous C_{60} electron transport layer and c) and d) showing EDX mappings of the lead Mα and iodide Mζ signal without any indication of PbI_2 particles in the bulk.

Additionally, **Figure S3** shows a SE image of the cross section of a 160 °C thin film sample with an extensive view on the THP surface which shows a distinct roughness but no sharp crystallite spikes which could penetrate the C_{60} layer or avoid a full coverage by the ETL layers.

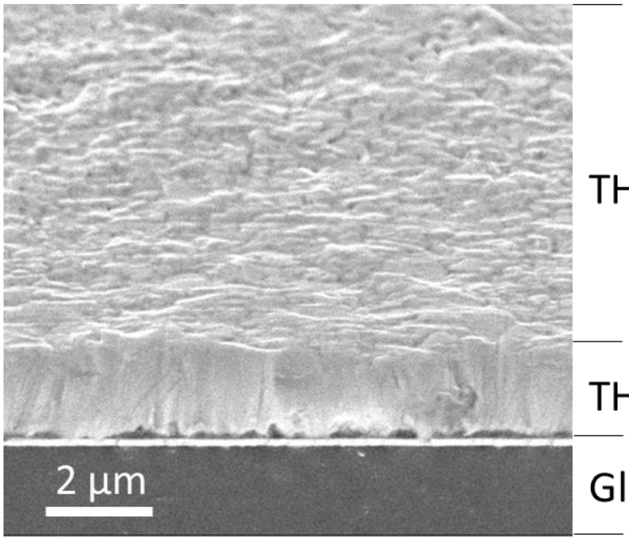

Figure S3: SE image of a thin-film sample (160 °C) without top electrode showing a subtle roughness of the top surface.

To confirm the existence of both, the THP and PbI_2 phases and to determine the wavelength of their CL intensity peak, CL spectra acquisitions were performed (**Figure S4**). The position of peaks is important for the selection of the right filters to selectively measure just one of the phases at a time. For the THP signal peak at 734 nm a 750 ± 50 nm band-pass filter was selected and for the 511 nm PbI_2 peak a 500 ± 50 nm band-pass filter.

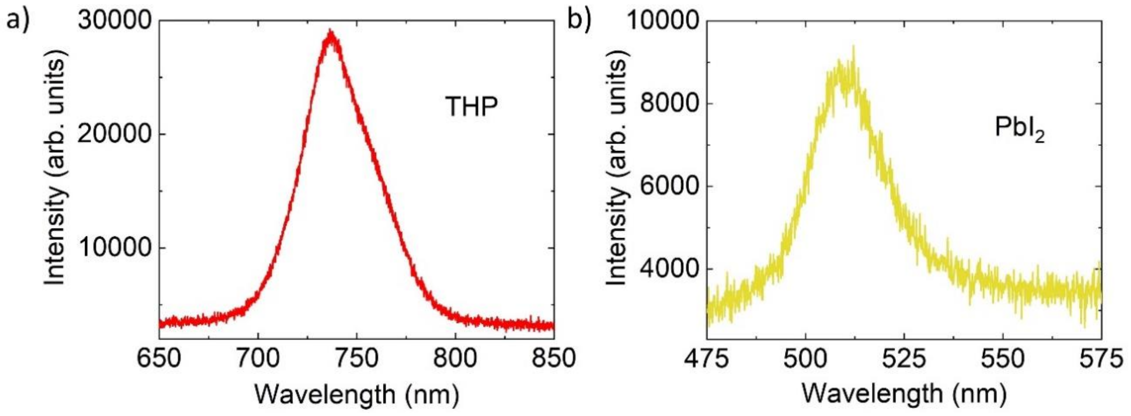

Figure S4: CL spectra showing peaks of a) THP at 734 nm and b) PbI_2 at 511 nm. This exhibits that 750 ± 50 nm and 500 ± 50 nm band-pass filters are suitable to selectively measure both types of signals without interfering with each other.

Xu et al. [1] suggested that the amount of chlorine in the thin film decreases with increasing annealing temperatures. EDX measurements indicated a chlorine loss at elevated temperatures. As a result, the band-gap energy can be expected to decrease. The decreasing peak energy of PL measurements in **Figure S5a** can be a result of decreasing band-gap energies and a further indication for chlorine loss. Such a decrease in band-gap energy can be expected to lead to a decrease of the V_{oc} of the corresponding solar cells. However, the decrease of almost 20 meV in band-gap energy between 100 and 170 °C cannot fully explain the measured decrease of V_{oc}. PbI_2 is still seen as the main driver of the V_{oc} loss. The PL quantum yield (PLQY) in **Figure S5b** reveals thin film crystallization issues in the 100 °C samples with the lowest PLQY even without being affected by PbI_2. This is discussed in the main article.

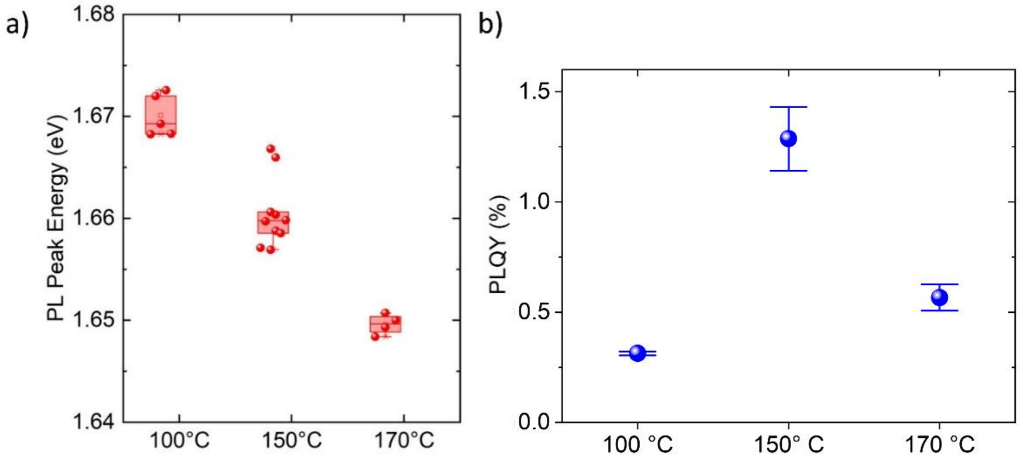

Figure S5: a) PL peak energies as a function of annealing temperature show a decrease of more than 20 meV. b) The PL quantum yield of the samples annealed at 100, 150 and 170 °C.

The *J-V* curves of the characterized solar cells were used to extract the series and shunt resistances, where the series resistances are shown in linear scales and the shunt resistances in logarithmic scales in the box plots in **Figure S6**. The median series resistances reveal a two-fold increase from 125 to 170 °C with significant consequences for the FF whereas the shunt resistances reveal median values from 0.6 to 2.4 kΩ·cm² which does not indicate any significant shunting issues with a corresponding impact on the solar-cell parameters.

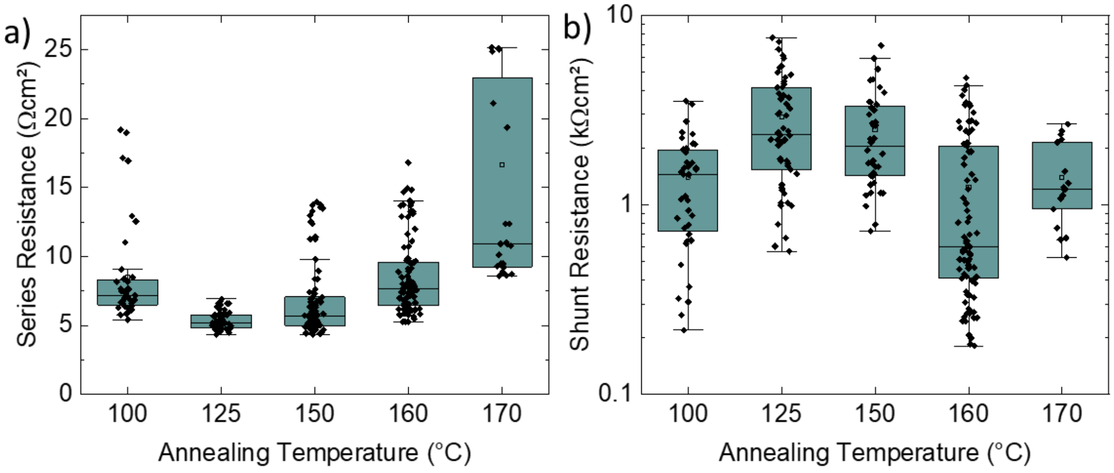

Figure S6: a) Series (linear) and b) shunt resistances (logarithmic) of the characterized solar cells as a function of annealing temperature.

Transient photoluminescence (TRPL) measurements were conducted (**Figure S7**) and analyzed by the bi-exponential model to extract the effective lifetimes t_{eff} for each sample. The results in **Table S2** reveal no clear trend and effective lifetimes in the same order of magnitude with a mean effective lifetime of 300 ± 100 ns. This agrees with our assumption that the detrimental effect such as increased recombination rates originates in the THP/C_{60} interface in combination with PbI_2 and not in the THP bulk. Since the thin film samples measured by TRPL do not have a C_{60} layer, the increased recombination rates with the consequence of decreased lifetimes cannot be shown.

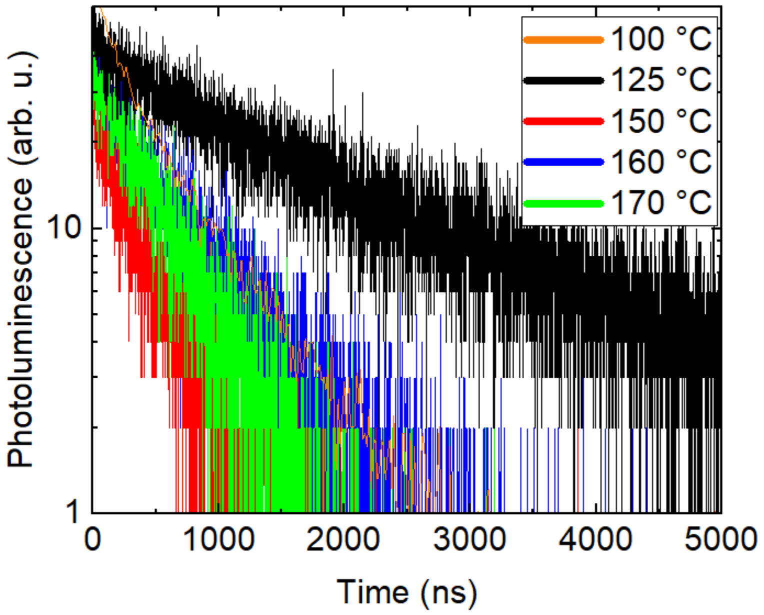

Figure S7: Logarithmic plots of the TRPL measurements of the THP thin-film samples

Table S2: The extracted effective lifetimes t_{eff} from TRPL measurements determined by means of a bi-exponential model.

**Sample**	t_{eff} **(ns)**
**100 °C**	240 ± 36
**125 °C**	540 ± 81
**150 °C**	130 ± 20
**160 °C**	290 ± 44
**170 °C**	270 ± 41

XRD patterns (**Figure S8a**) measured at an incident X-ray beam angle of 2° to enhance the surface sensitivity showed an increasing amount of PbI_2 by an increasing intensity of the 001 PbI_2

diffraction peaks. Furthermore, the full width of half maximum (FWHM) of the 100 THP Bragg peaks were extracted and plotted in **Figure S8b** exhibiting a decreasing FWHM with increasing annealing temperature. According to the Scherrer equation, a diffraction peak broadening can imply a decrease of grain sizes.

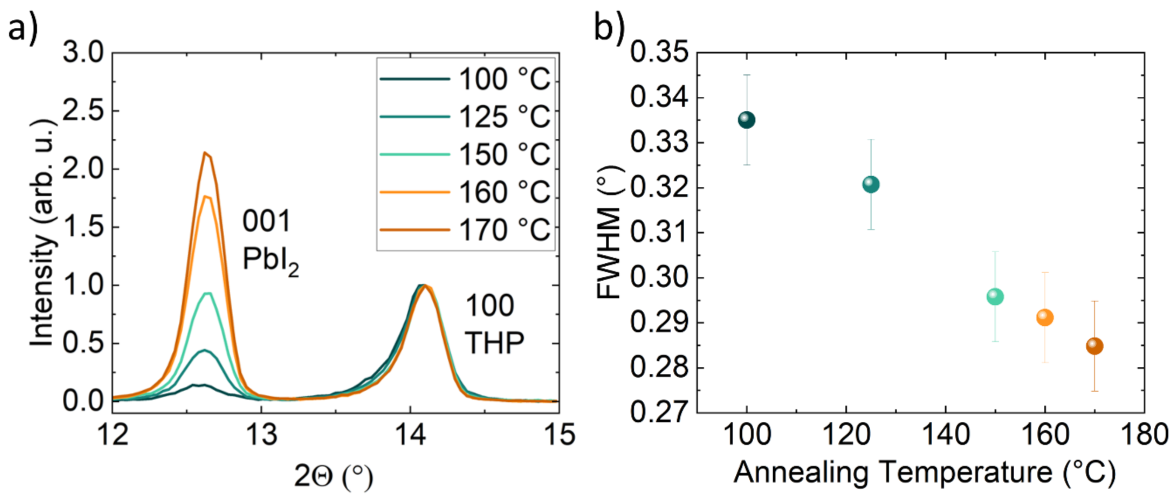

Figure S8: a) XRD pattern with normalized 100 THP Bragg peak exhibits a decreasing intensity of the 001 PbI_2 Bragg peak when varying the annealing temperature from 100 to 170 °C. The decreasing 100 THP Bragg peak width, shown in b), can indicate increasing grain sizes with increasing annealing temperature.

The correlation between PbI_2 coverage and shunt resistance, depicted in **Figure S9** is less evident, since beside the typical outlier 100 °C data point, 160 °C seems out of the order as well. Analogous to the analysis of the series resistances a hypothetical PbI_2-free shunt resistance of 2.7 kΩ·cm² was determined. But if we apply the model with Green's approximation analogous to the case of the series resistance the impact on the power conversion efficiencies for a complete PbI_2 removal is almost an order of magnitude lower compared with the series resistance impact. The largest effect, a 1% increase of PCE, can be seen for the 160 °C which seemingly has some shunt resistance issues.

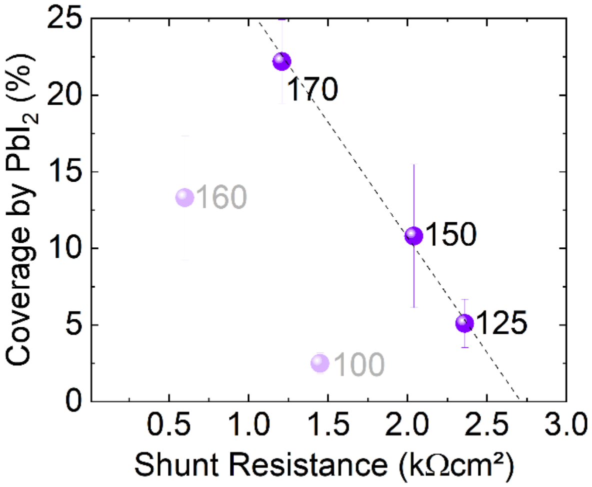

Figure S9: The estimated PbI_2 coverage as a function of the median shunt resistances with a guide for the eyes (dashed line) showing a theoretical PbI_2-free shunt resistance of 2.7 kΩ·cm² at the x-intercept. The measurement points of the 100 and 160 °C samples are included in a weaker intensity to highlight them as outliers.

REFERENCES

(1) Xu, K.; Al-Ashouri, A.; Peng, Z. W.; Köhnen, E.; Hempel, H.; Akhundova, F.; Marquez, J. A.; Tockhorn, P.; Shargaieva, O.; Ruske, F.; Zhang, J.; Dagar, J.; Stannowski, B.; Unold, T.; Abou-Ras, D.; Unger, E.; Korte, L.; Albrecht, S. Slot-Die Coated Triple-Halide Perovskites for Efficient and Scalable Perovskite/Silicon Tandem Solar Cells. *ACS Energy Lett.* **2022**, *7* (10), 3600–3611. https://doi.org/10.1021/ACSENERGYLETT.2C01506.
